# MEGF10, a Glioma Survival-Associated Molecular Signature, Predicts IDH Mutation Status

**DOI:** 10.1155/2018/5975216

**Published:** 2018-05-20

**Authors:** Guanzhang Li, Zhiliang Wang, Chuanbao Zhang, Xing Liu, Fuqiang Yang, Lihua Sun, Jingshan Liang, Huimin Hu, Yanwei Liu, Gan You, Zhaoshi Bao, Wei Zhang, Zheng Wang, Tao Jiang

**Affiliations:** ^1^Department of Molecular Neuropathology, Beijing Neurosurgical Institute, Capital Medical University, Beijing, China; ^2^Department of Neurosurgery, Beijing Tiantan Hospital, Capital Medical University, Beijing, China; ^3^Center of Brain Tumor, Beijing Institute for Brain Disorders, Beijing, China; ^4^China National Clinical Research Center for Neurological Diseases, Beijing, China; ^5^Chinese Glioma Genome Atlas Network (CGGA), Beijing, China

## Abstract

Glioma is the most common primary brain tumor with various genetic alterations; among which, IDH mutation is the most common mutation and plays an important role in glioma early development, especially in lower grade glioma (LGG, WHO II-III). Previous studies have found that IDH mutation is tightly associated with extensive methylation across whole genome in glioma. To further investigate the role of IDH, we obtained methylation data of 777 samples from CGGA (Chinese Glioma Genome Atlas) and TCGA (The Cancer Genome Atlas) with IDH mutation status available. A package compiled under R language called *Tspair* was used as the main analytic tool to find potential probes that were significantly affected by IDH mutation. As a result, we found one pair of probes, *cg06940792* and *cg26025891*, which was capable of predicting IDH mutation status precisely. The hypermethylated probe was *cg06940792*, designed in the promoter region of *MEGF10*, while the hypomethylated probe was *cg26025891*, designed in the promoter region of *PSTPIP1*. Survival analysis proved that hypermethylation or low expression of *MEGF10* indicated a favorable prognosis in 983 glioma samples. Moreover, gene ontology analysis demonstrated that *MEGF10* was associated with cell migration, cell proliferation, and regulation of apoptosis in glioma. All findings above can be validated in three other independent cohorts. In a word, our results suggested that methylation level and mRNA expression of *MEGF10* in glioma were not only correlated with IDH mutation but also associated with clinical outcome of patients, providing potential guide for future dissection of IDH role in glioma.

## 1. Introduction

Glioma is the most common primary brain tumor with various genetic alterations and dismal outcomes [1, 2]. Despite aggressive surgical resection followed by concomitant chemoradiotherapy and/or adjuvant chemotherapy, the prognosis of these patients remains poor. Along with WHO grade, extent of tumor resection, and Karnofsky performance status (KPS) score, IDH mutation is one of the most robust prognosticators for glioma patients. In the past decades, various biomarkers have been reported to be associated with malignant phenotype of glioma and prognosis [3]. Somatic mutations in IDH can be detected in 68%–80% LGG patients [4]. Glioma with IDH mutation shows distinct genetic and clinical patterns from those with wild-type IDH [5]. Due to high frequency and widespread impact on tumor genome, IDH mutation has been proposed to be one of the initiators of glioma [6, 7]. However, the mechanism of IDH in glioma remains an enigma. As previously reported, IDH mutation induces increased methylation of numerous genes, including many oncogenes.

In the present study, we collected 24 glioblastoma (GBM) samples with methylation microarray data available and 502 samples with mRNA microarray data from CGGA and TCGA. With *Tspair* package, we asked the correlation between IDH mutation status and methylation levels of whole genome gene in LGG and GBM samples and revealed that probe *cg06940792* of *MEGF10* together with probe *cg26025891* of *PSTPIP1* was the best paired probe to predict IDH mutation status. Hypermethylation or low expression of *MEGF10* indicated a favorable prognosis by Kaplan-Meier survival analysis. GSVA analysis proved that *MEGF10* plays an important role in cell migration, apoptosis, and proliferation. Moreover, these findings were further validated on another two independent validation cohorts. Overall, our results provided novel insights into *MEGF10*, an important IDH mutation predictor, and provided valuable reference for future IDH mechanism deciphering.

## 2. Methods

### 2.1. Patients and Samples

DNA methylation profile of GBM samples was from the Chinese Glioma Genome Atlas (CGGA), generated by Illumina Infinium Human Methylation 27K Bead Chip. Patients were eligible for the study if their diagnosis was established histologically by 2 neuropathologists according to the 2007 WHO classification guidelines. Only samples with more than 80% tumor cells were selected. This study was approved by the institutional review board (IRB) of Beijing Tiantan Hospital. Written consent was obtained from each patient. The independent sample cohorts of The Cancer Genome Atlas (TCGA) and GSE16011 databases were downloaded from public databases (http://www.cgga.org.cn, http://cancergenome.nih.gov/ and http://www.ncbi.nlm.nih.gov/geo/query/acc.cgi?acc=GSE16011).

### 2.2. IDH Mutation

Genomic DNA was isolated from frozen tissues with a QIAamp DNA Mini Kit (Qiagen) as the manufacturer's protocol. Pyrosequencing of IDH1/2 mutations was supported by Gene-tech (Shanghai, China). The primers 5′-GCTTGTGAGTGGATGGGTAAAAC-3′, 5′-BiotinTTGCCAACATGAC TTACTTGATC-3′ for IDH1 and 5′-ATCCTGGGGGGGACTGTCTT-3′, 5′-Biotin-CTCTCCACCCTGGCCT ACCT-3′ for IDH2 were used for PCR amplification, and the primers 5′-TGGATGGGTAAAACCT-3′ for IDH1 and 5′-AGCCCATCACCATTG-3′ for IDH2 were used for pyrosequencing.

### 2.3. Gene Set Variation Analysis (GSVA)

After Spearman correlation analysis, we found that certain MEGF10-related genes were enriched in particular gene ontology terms or KEGG pathways; subsequent heatmaps were generated according to pathway or GO terms with GSVA package developed by Hanzelmann et al. [8]. Gene ontology (GO) and KEGG pathway gene sets were downloaded from a public database (http://amigo.geneontology.org/amigo/landing).

### 2.4. Statistical Analysis

*Top scoring pair* (*Tspair)* package developed by Leek [9] was used as the main tool to find methylation probe pairs that was able of predicting IDH mutation status. The specificity and sensitivity of candidate pair methylation probes were determined by receiver operating characteristic (ROC) analysis with *pROC* package developed by Robin et al. [10]. Overall survival was estimated from the date of diagnosis to the date of either death or last follow-up. Patients were censored at the time they were last known to be alive. Kaplan-Meier analysis was performed to estimate the survival time of different subgroups. *p* value < 0.05 was considered to be statistically significant. All statistical computations were performed with the statistical software environment R, version 3.1.0 (http://www.r-project.org/) or GraphPad Prism version 6.01.

## 3. Results

### 3.1. Methylation Level of MEGF10 Is an IDH Mutation Predictor with the Superb Specificity in Glioma

**Figure 1:**
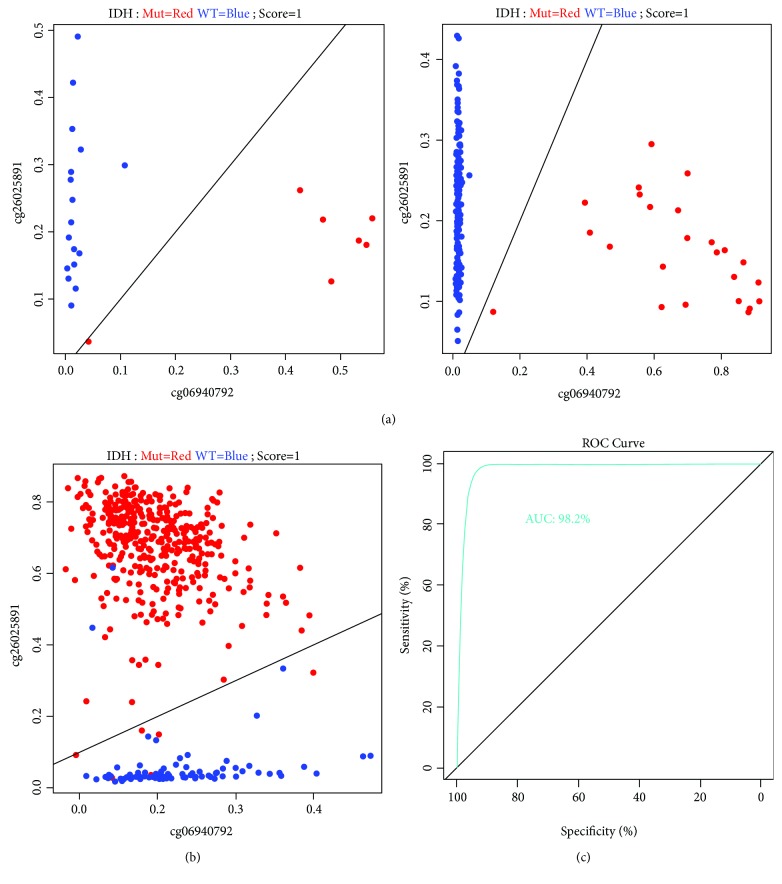
Methylation levels of *MEGF10* have high efficiency to predict IDH mutation. (a) *Tspair* analysis showed that IDH mutation status can be predicted by methylation levels of cg06940792 and cg26025891 in GBM patients. (b, c) *Tspair* and ROC curve analysis confirmed the high performance of candidates as a predictor of IDH mutation in LGG patients.

To identify candidate probes that can predict IDH mutation, we compared gene methylation levels between IDH mutation and wild-type IDH in 24 GBM samples by *Tspair* analysis, a method for calculating the top scoring pair for classification of high-dimensional data sets. A total of 21,286 probe pairs were identified as top scoring pairs. Additionally, we identified 4137 probe pairs in TCGA GBM methylation microarray dataset with the same method. Then, we overlapped the candidate probes and finally validated 2 pairs of probes—*cg06940792* versus *cg26025891* and *cg11465971* versus *cg18342900* (Figure 1(a)). We projected these probes into TCGA LGG patients as validation, and only the first pair, *cg06940792* versus *cg26025891*, showed consistent prediction value for IDH mutation (Figure 1(b)). Therefore, in TCGA dataset, the methylation levels of *MEGF10* (*cg06940792*) had the best specificity to predict IDH mutation status (Figure 1(c)).

### 3.2. Hypermethylation and Low mRNA Expression of MEGF10 Confer Improved Overall Survival in Glioma

**Figure 2:**
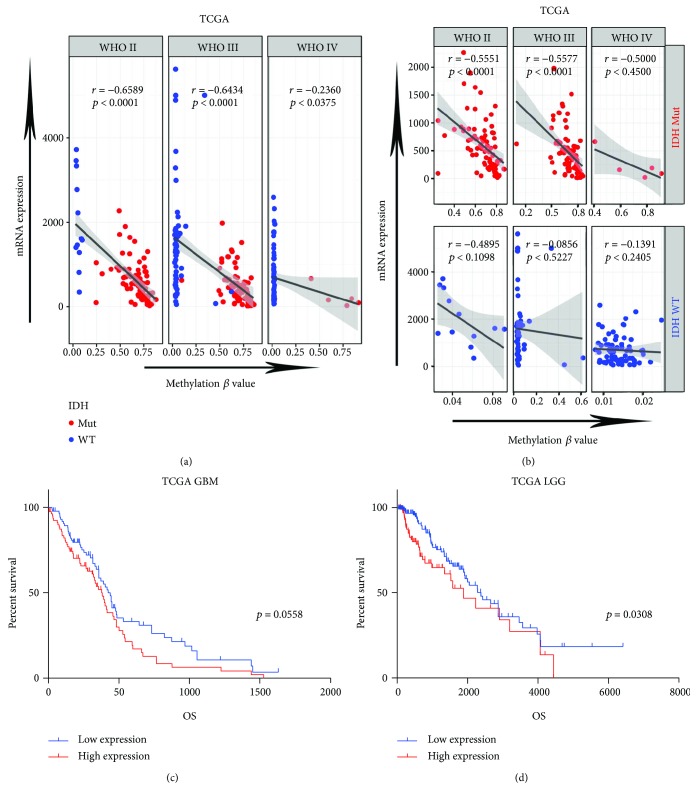
*MEGF10* mRNA expression was related to clinical outcomes in gliomas. (a) By Spearman correlation analysis, mRNA expression of *MEGF10* was negatively correlated with methylation levels in different WHO grades of glioma. (b) Spearman correlation analysis revealed the correlations between methylation levels and expressions in different WHO grades and different IDH mutation statuses. The correlation was affected by IDH mutation status in all WHO grades. (c, d) Kaplan-Meier estimates of survival for 407 patients in TCGA with RNA-seq. There is a significant difference in survival between *MEGF10* high-expression and low-expression patients (LGG, *p* < 0.05; GBM, *p* < 0.05).

Increasing evidence has shown that hypermethylation of genes caused by IDH mutation usually results in downregulation of expression. To evaluate the impact that methylation exerts on expression of *MEGF10*, we investigated the relationship between gene expression and methylation levels in both TCGA transcriptome sequencing (RNA-seq) dataset (239 LGG, 168 GBM) and methylation microarray dataset (527 LGG, 295 GBM) by the Spearman correlation test. We found that methylation level of *MEGF10* was strongly negatively correlated with mRNA expression in hypermethylated patients, but showed very limited relevance in hypomethylated patients (Figure 2(a)). It is noteworthy that the correlations between them seem to be significantly affected by IDH mutation status. Therefore, these patients were subsequently divided into two groups based according to IDH mutation status and were analyzed again. Interestingly, robust relevance was observed between *MEGF10* methylation and mRNA in IDH mutation group, but not in wild-type group (Figure 2(b)). We noticed that in the IDH mutation group *p* value of the Spearman correlation was >0.05. This may be accounted for by a limited sample size. Kaplan-Meier survival analysis showed that low expression of *MEGF10* conferred a longer overall survival (Figure 2(c)). These results were further validated in CGGA and GSE16011 mRNA microarray cohorts (Figure S1).

### 3.3. Independence of MEGF10 from the Traditional Clinical and Pathological Factors in Glioma

**Figure 3:**
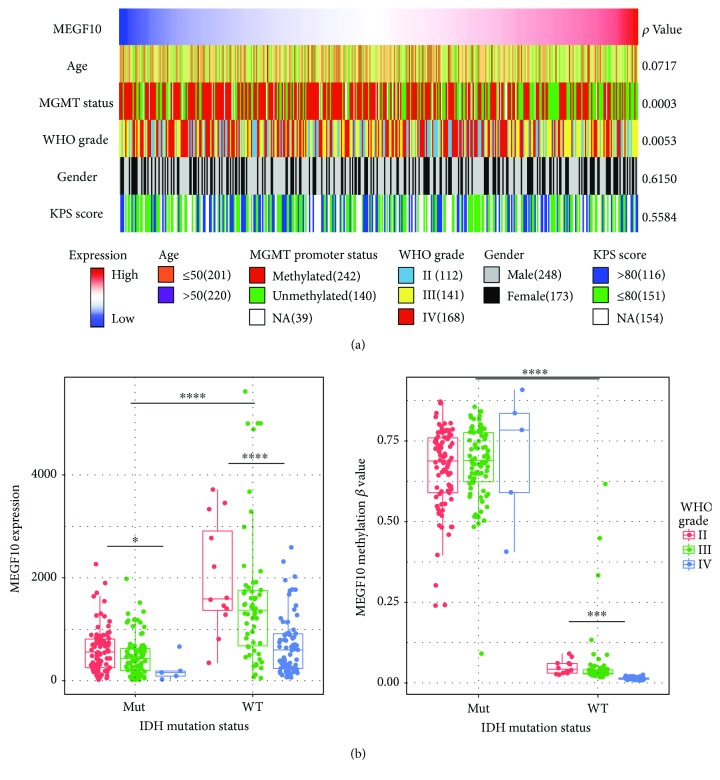
*MEGF10* associated clinical and molecular pathological factors in TCGA. (a) *MEGF10* expression has significant correlation with MGMT promoter methylation status and WHO histological grade. However, it has no significant correlation with age, gender, and preoperative Karnofsky performance status (KPS) score. (b) *MEGF10* expression and methylation *β* value have significant correlation with IDH mutation status with different WHO histological grades. ^∗^ *p* < 0.05, ^∗∗∗^ *p* < 0.0005, and ^∗∗∗∗^ *p* < 0.0001.

To confirm *MEGF10* as an independent predictor with previous widely accepted factors (age, WHO histological grade, and MGMT promoter status), we collected and analyzed the corresponding clinical and molecular information of glioma patients from TCGA datasets. As was shown in Figure 3, *MEGF10* expression showed significant correlation with MGMT promoter methylation status, IDH mutation status, and WHO histological grade. Moreover, we further performed univariate and multivariate Cox regression analyses in the TCGA glioma cohort. On univariate analysis, *MEGF10* was significantly associated with survival (*p* = 0.047) along with age at diagnosis, WHO histological grade, and MGMT promoter status. On multivariate analysis, the risk score was still significant (*p* = 0.036) after adjusting for patient age at diagnosis, WHO histological grade, and MGMT promoter status (Table 1).

### 3.4. MEGF10 Affects Migration, Proliferation, and Apoptosis of Glioma Cells

**Figure 4:**
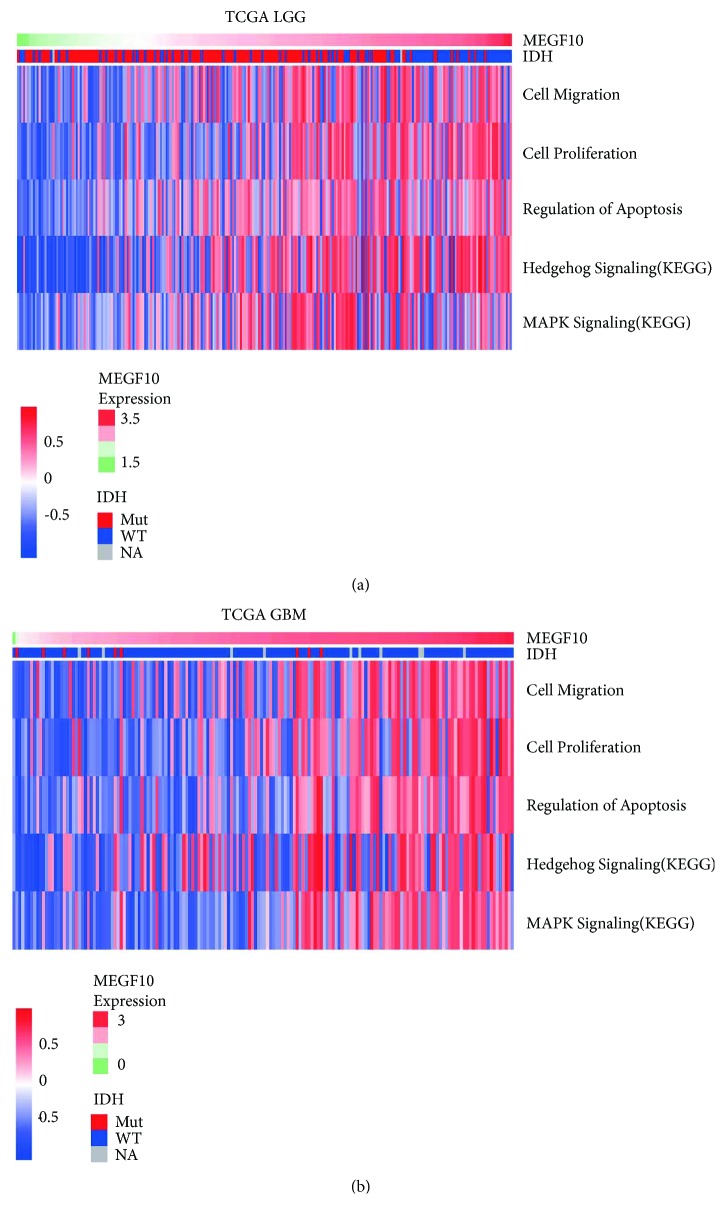
*MEGF10* associated genes and their potential functions. (a) Gene set variation analysis of biofunction (migration, proliferation, and apoptosis) and signaling pathway (Hedgehog signaling and MAPK signaling) associated genes in TCGA LGG RNA-seq patients. Gene set enrichment score was analyzed by GSVA package of R. These genes showed higher expression with the *MEGF10* expression going from low to high. (b) Above results could be validated in TCGA GBM RNA-seq cohorts.

To examine functional effects of *MEGF10* on glioma cells, Spearman correlation analysis was performed with TCGA GBM RNA-seq dataset. In total, 1124 genes were found to be most positively correlated with *MEGF10* (*r* > 0.3). Among these genes, 30 of them were validated as functionally related genes. Through Gene Set Variation Analysis (GSVA) of these functional genes, we obtained biological roles of *MEGF10*. As an oncogene, *MEGF10* promoted migration and proliferation of glioma cells as well as taking part in cell apoptosis regulation through Hedgehog signaling and MAPK signaling (Figure 4(a)). As expected, we observed similar results in LGG patients (Figure 4(b)). These results were further validated in another two independent validation cohorts mentioned above (Figure S2).

## 4. Discussion

Various genetic alterations and disordered gene functions are distinguishing features of glioma [3, 11]. IDH mutation, as a high-frequency mutation, makes an increasing sense since discovered [12]. It is generally accepted that IDH mutation plays an important role in early glioma development and predicts improved clinical outcomes compared with wild-type IDH [5, 13]. However, the mechanism of IDH remains to be elucidated. Previous researches show that IDH mutation is closely related to glioma CpG island methylator phenotype (G-CIMP^+^) [14, 15]. Hypermethylation of some CpG islands in cancer has been reported to be associated with silencing of oncogenes or tumor suppressor genes [16, 17]. As DNA methylation is a highly stable process and can inherit epigenetic pattern for several cell generations, it has been studied thoroughly in glioma recently [18, 19]. In this study, we use gene methylation level to predict IDH mutation status in glioma for the first time. Thus, this finding will aid in deciphering the function of IDH in brain tumors.

*Tspair* is a simple and stable classification method with perfect accuracy and has been successfully applied in other tumors [20]. Here, we analyzed the genome-wide methylation microarray to explore potential specific predictor for IDH mutation. Fortunately, we obtained *MEGF10* (*cg06940792*), the best candidate that could be used to predict IDH mutation status accurately in LGG and GBM patients. Moreover, hypermethylation of this candidate also downregulated corresponding gene expression. Interestingly, we find that the relation between *MEGF10* expression and methylation level is closely related to IDH mutation status. These findings prompt that expression of *MEGF10* is downregulated by the corresponding hypermethylation, which is most likely caused by IDH mutation.

*MEGF10*, multiple EGF-like domains 10, is a member of the multiple epidermal growth factor-like domains protein family. This scant concerned protein plays a role in cell adhesion, motility, and proliferation and is a mediator of apoptosis as described in NCBI gene summary (http://www.ncbi.nlm.nih.gov/gene/84466). Early studies showed that it plays a significant role in nerve system development and metabolism in the brain [21, 22]. To date, *MEGF10* has not been reported to be involved in malignant tumors. To verify the conjecture that *MEGF10*, as a prognostic factor, plays a potential role in glioma tumorigenesis, Kaplan-Meier survival analysis, univariate and multivariate Cox regression analyses, and GSVA analysis are performed. Finally, *MEGF10* is found to be a prognostic factor and plays a potential role in tumorigenesis. It is worth noting that high expression of *MEGF10* predicts hypomethylated MGMT promoter, which leads to high expression of MGMT and temozolomide (TMZ) resistance. Despite adequate statistics proven among three public databases, experimental validation needs to be further perfected.

In conclusion, our findings shed a new light on understanding the fundamental basis of IDH mutation in glioma. Most importantly, *MEGF10* is a promising novel target that warranted further investigation.

## Figures and Tables

**Table 1:** Univariate and multivariate Cox regression analyses in TCGA glioma samples.

Variable	Univariate analysis				Multivariate analysis			
	HR	HR (95% CI)		*p* value	HR	HR (95% CI)		*p* value
		Lower	Upper			Lower	Upper	
Histological grade (WHO)	7.337	5.241	10.273	<0.0001	3.486	2.358	5.154	<0.0001
Age	1.069	1.057	1.082	<0.0001	1.048	1.035	1.062	<0.0001
MGMT promoter status	0.353	0.265	0.471	<0.0001	0.632	0.46	0.868	0.005
MEGF10 expression	1.362	1.004	1.848	0.047	1.412	1.022	1.951	0.036

## Supplementary Materials

Supplementary Materials Supplementary Figure 1: MEGF10 mRNA expression was related to clinical outcomes in CGGA and GSE16011 cohorts. A/B Kaplan-Meier survival analysis showed that low expression of MEGF10 conferred a longer overall survival in CGGA microarray (LGG: *p* < 0.05, GBM: *p* < 0.05). C/D Above results could be validated in GSE16011 microarray (LGG: *p* < 0.05, GBM: *p* < 0.01). Supplementary Figure 2: GSVA analysis of MEGF10 associated functional genes in CGGA microarray and GSE16011 microarray cohorts. A/B GSVA analysis of MEGF10 associated functional genes in CGGA microarray (LGG: A, GBM: B). C/D Above results further validated in GSE16011 microarray (LGG: C, GBM: D).
