# Supplementary material for: MEGF10, a Glioma Survival-Associated Molecular Signature, Predicts IDH Mutation Status
Source: Dis Markers. 2018 May 20;2018:5975216. doi: 10.1155/2018/5975216 (PMC5985127; doi:10.1155/2018/5975216)
Supplement: Supplementary Materials — Supplementary Figure 1: MEGF10 mRNA expression was related to clinical outcomes in CGGA and GSE16011 cohorts. A/B Kaplan-Meier survival analysis showed that low expression of MEGF10 conferred a longer overall survival in CGGA microarray (LGG: p < 0.05, GBM: p < 0.05). C/D Above results could be validated in GSE16011 microarray (LGG: p < 0.05, GBM: p < 0.01). Supplementary Figure 2: GSVA analysis of MEGF10 associated functional genes in CGGA microarray and GSE16011 microarray cohorts. A/B GSVA analysis of MEGF10 associated functional genes in CGGA microarray (LGG: A, GBM: B). C/D Above results further validated in GSE16011 microarray (LGG: C, GBM: D). [file 5975216.f1.zip › figure S1(ppt)_DM_2255429.pptx]

## Slide 1
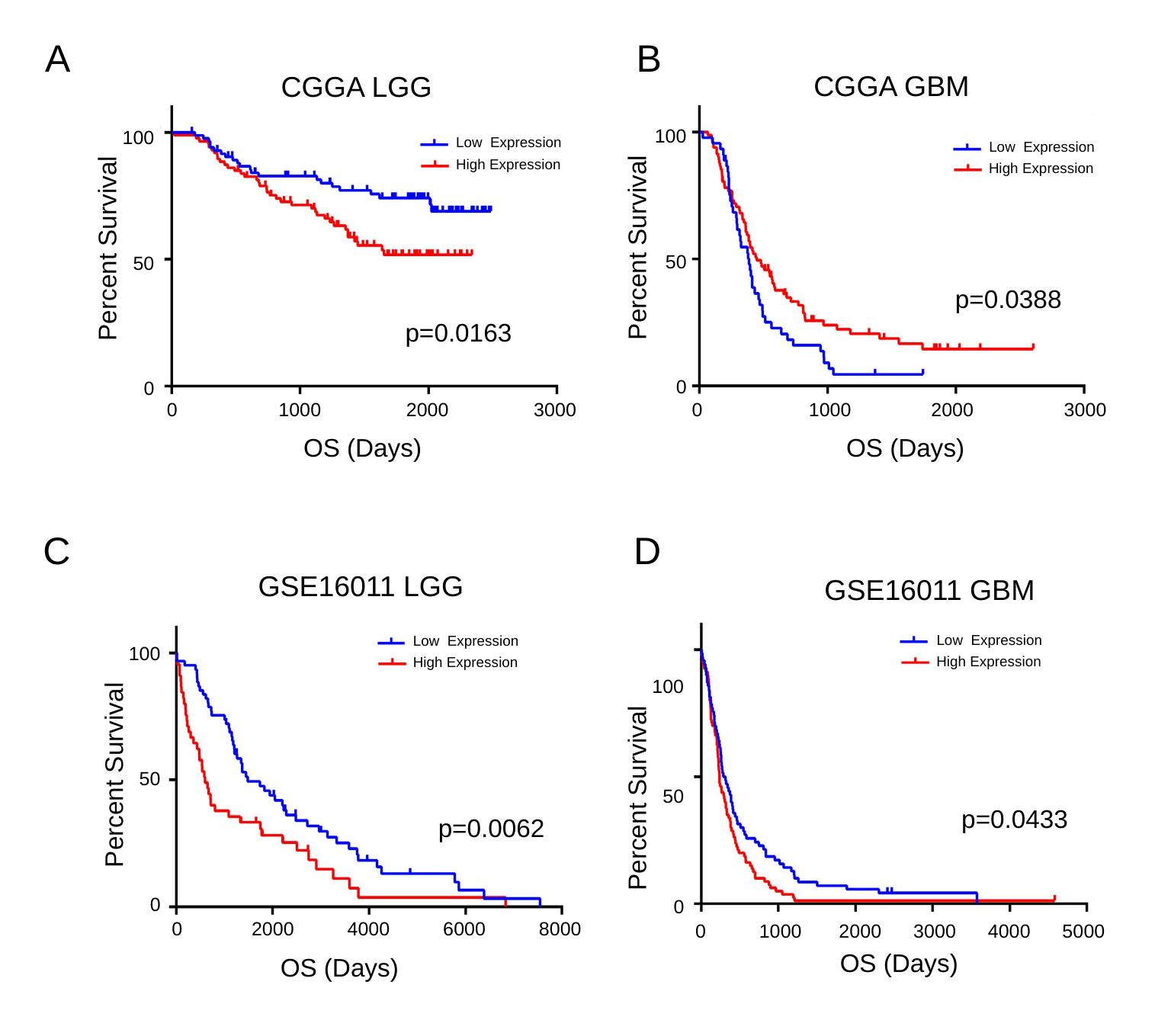

B
A
CGGA GBM
CGGA LGG
100
50
0
100
50
0
Low Expression
Low Expression
High Expression
Percent Survival
High Expression
Percent Survival
p=0.0388
p=0.0163
0 1000 2000 3000
0 1000 2000 3000
OS (Days)
OS (Days)
D
C
GSE16011 LGG
GSE16011 GBM
Low Expression
Low Expression
100
50
0
High Expression
High Expression
100
50
0
Percent Survival
Percent Survival
p=0.0433
p=0.0062
0 2000 4000 6000 8000
0 1000 2000 3000 4000 5000
OS (Days)
OS (Days)
