# Supplementary material for: MEGF10, a Glioma Survival-Associated Molecular Signature, Predicts IDH Mutation Status
Source: Dis Markers. 2018 May 20;2018:5975216. doi: 10.1155/2018/5975216 (PMC5985127; doi:10.1155/2018/5975216)
Supplement: Supplementary Materials — Supplementary Figure 1: MEGF10 mRNA expression was related to clinical outcomes in CGGA and GSE16011 cohorts. A/B Kaplan-Meier survival analysis showed that low expression of MEGF10 conferred a longer overall survival in CGGA microarray (LGG: p < 0.05, GBM: p < 0.05). C/D Above results could be validated in GSE16011 microarray (LGG: p < 0.05, GBM: p < 0.01). Supplementary Figure 2: GSVA analysis of MEGF10 associated functional genes in CGGA microarray and GSE16011 microarray cohorts. A/B GSVA analysis of MEGF10 associated functional genes in CGGA microarray (LGG: A, GBM: B). C/D Above results further validated in GSE16011 microarray (LGG: C, GBM: D). [file 5975216.f1.zip › figure S2(ppt)_DM_2255430.pptx]

## Slide 1
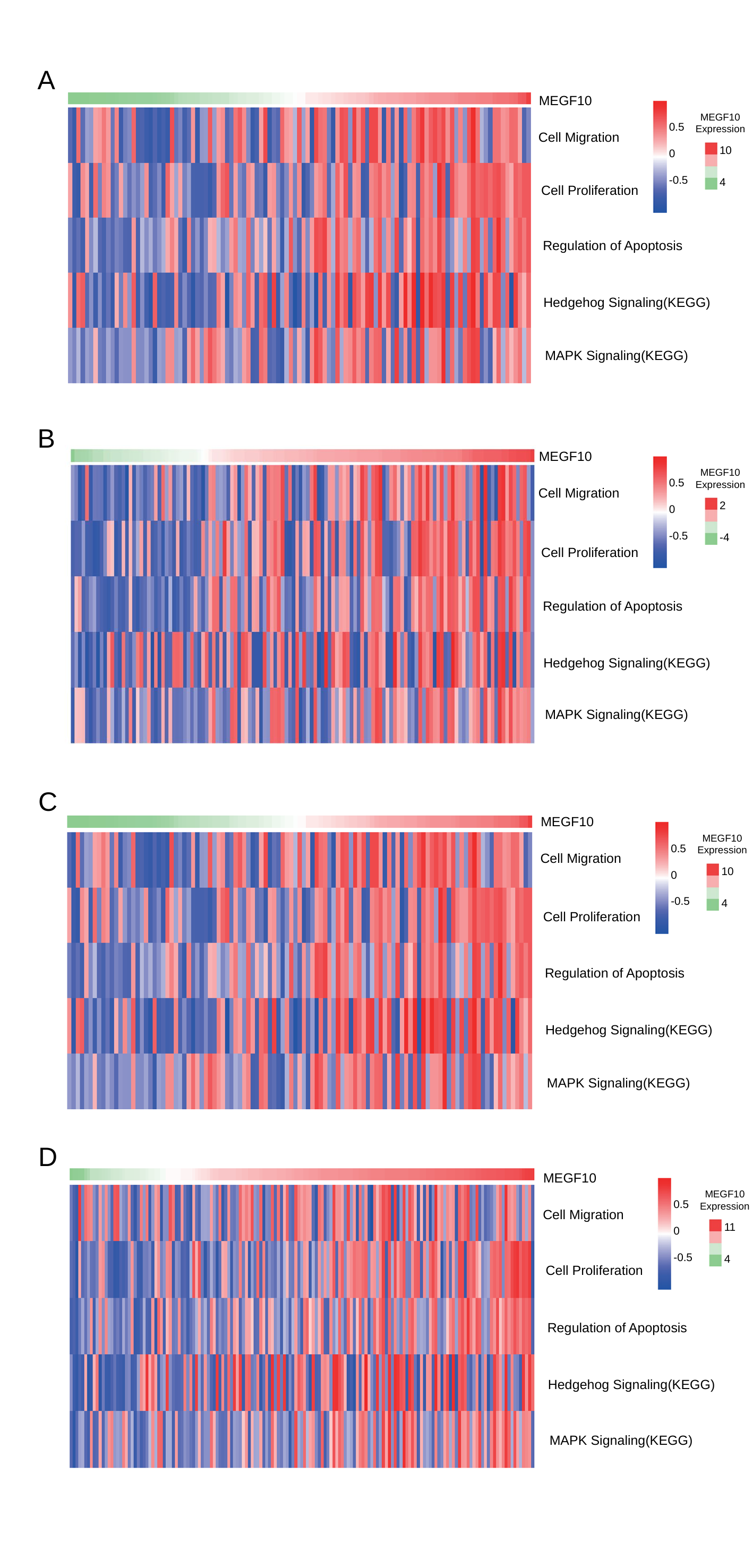

A
MEGF10
MEGF10
Expression
0.5
0
-0.5
Cell Migration
10
4
Cell Proliferation
Regulation of Apoptosis
Hedgehog Signaling(KEGG)
MAPK Signaling(KEGG)
B
MEGF10
MEGF10
Expression
0.5
0
-0.5
Cell Migration
2
-4
Cell Proliferation
Regulation of Apoptosis
Hedgehog Signaling(KEGG)
MAPK Signaling(KEGG)
C
MEGF10
MEGF10
Expression
0.5
0
-0.5
Cell Migration
10
4
Cell Proliferation
Regulation of Apoptosis
Hedgehog Signaling(KEGG)
MAPK Signaling(KEGG)
D
MEGF10
MEGF10
Expression
0.5
0
-0.5
Cell Migration
11
4
Cell Proliferation
Regulation of Apoptosis
Hedgehog Signaling(KEGG)
MAPK Signaling(KEGG)
